# Pulmonary Rehabilitation of a Case With Worsening Dyspnea Due to Acute Exacerbation of Interstitial Pneumonia After Video-Assisted Thoracic Surgery

**DOI:** 10.7759/cureus.22545

**Published:** 2022-02-23

**Authors:** Kohji Iwai, Jun Hanaoka

**Affiliations:** 1 Division of Physical Therapy, Faculty of Rehabilitation and Care, Seijoh University, Tokai, JPN; 2 Department of Thoracic Surgery, Shiga University of Medical Science, Otsu, JPN

**Keywords:** exercise capacity, pulmonary rehabilitation, acute exacerbation, interstitial pneumonia, video-assisted thoracic surgery

## Abstract

We report a case of acute exacerbation of interstitial pneumonia after video-assisted thoracic surgery. The case was a 69-year-old man with left upper lobe lung cancer. Acute exacerbation was suspected on postoperative day 6 due to worsening dyspnea and frosted shadows on computed tomography. High-dose corticosteroids and low-to-moderate-dose corticosteroids were administered. Step-by-step rehabilitation with oxygen administration commenced as soon as possible, and the patient was able to be discharged. However, dyspnea, knee extension strength, and exercise capacity were significantly worse than before surgery. Eighteen months later, pulmonary function and knee extension strength showed improvements, but exercise capacity was unchanged from the time of discharge. Continued follow-up will be necessary.

## Introduction

Acute postoperative exacerbation of interstitial pneumonia (IP) is a serious complication following surgical treatment for lung cancer combined with IP [1]. Of patients undergoing surgical treatment for lung cancer in Japan, 5% have comorbid IP, of which 9.3% develop acute exacerbations. The mortality rate is 43.9% once an acute exacerbation occurs [2]. In other words, acute postoperative exacerbation of IP results in very high mortality, as only half of the patients survive. However, the early rehabilitation of patients with acute IP exacerbation after video-assisted thoracic surgery (VATS) lobectomy and its clinical course has not been reported. Herein, we report a case of acute exacerbation of IP and markedly worsening dyspnea after VATS lobectomy, who was discharged following early rehabilitation. A follow-up assessment was conducted 18 months later.

## Case presentation

A 69-year-old man with left upper lobe lung cancer had a preoperative diagnosis of cT1cN0M0 stage IA3. Height 179 cm, weight 67.1 kg, body mass index 21 kg/m^2^, 49 pack-years, and Mini-Mental State Examination 30/30 points. During a follow-up assessment at another hospital for pulmonary fibrosis with emphysema from 15 years ago, abnormal shadows were observed on computed tomography and were suspected of being primary lung cancer. A cytological diagnosis of adenocarcinoma was made, and the patient was admitted for surgery. The preoperative blood test results showed C-reactive protein; 0.2 mg/dL, white blood cells; 7,800 μL, sialylated carbohydrate antigen KL-6: 407 U/mL, hemoglobin; 15.4 g/dL, neuron-specific enolase; 12.4 ng/mL, sialyl Lewis-x antigen; 30 U/mL, pro-gastrin releasing peptide; 49.2 pg/mL, and cytokeratin 19 fragments; less than 1.0. The acute exacerbation risk score was 11 points with a predicted incidence of 10%-25% [3]. A preoperative evaluation was performed in the rehabilitation room one day before the left upper lobectomy.

**Figure 1:**
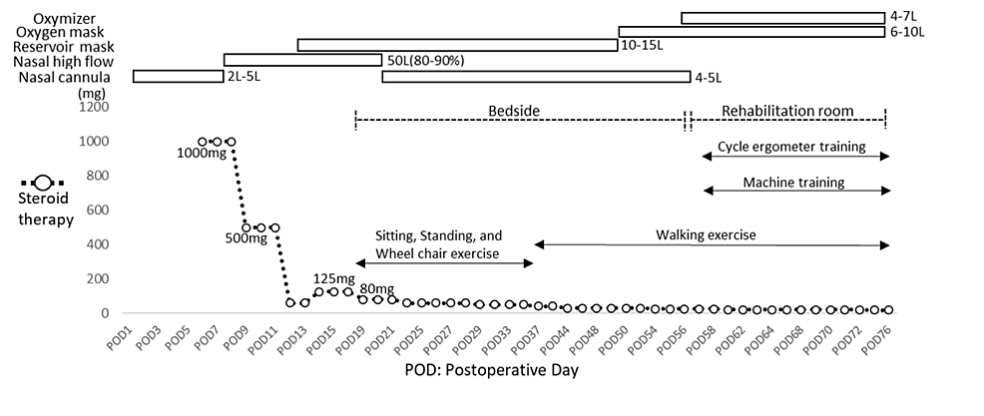
Clinical course; oxygen administration device, changes in steroid therapy, and early rehabilitation.

**Figure 2:**
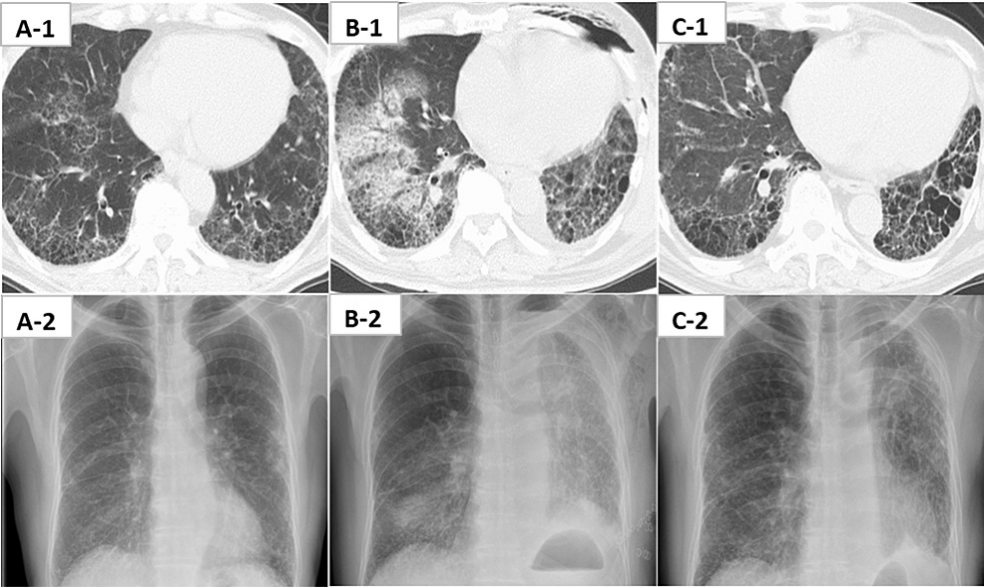
Demonstrable images of computed tomography (1) and chest x-ray (2). (A) Before surgery. (B) During acute exacerbation (POD6). (C) After 18 months. POD; postoperative day

The clinical course (oxygen administration device, steroid therapy, and early rehabilitation) is shown in Figure 1. Acute exacerbation was suspected on postoperative day (POD) 6 due to worsening dyspnea and frosted shadows (Figure 2). High-dose corticosteroids and low-to-moderate-dose corticosteroids were administered. Due to persistent feelings of severe dyspnea (modified Medical Research Council Dyspnea scale 4), early rehabilitation resumed from POD 18 with bedside rehabilitation, and sitting and standing exercises were performed while adjusting the oxygen device. Walking exercises commenced from POD 36. Training in the rehabilitation room started on POD 56, with cycle ergometer exercise and machine training (leg press) implemented from POD 58. Adjusting the exercise load was performed with careful attention to the patient’s feelings of dyspnea, leg fatigue using a modified Borg Scale (5 or less), and exercise-induced hypoxemia. Rehabilitation sessions were 20-40 minutes per day in duration, including rest periods.

**Figure 3:**
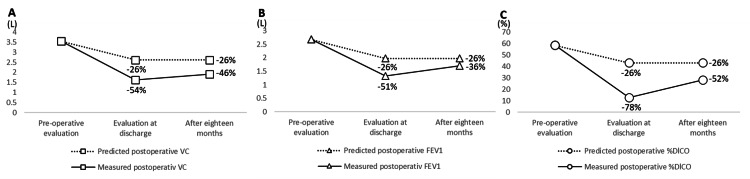
Predicted and measured vital capacity (A), forced expiratory volume in one second (B), and percentage diffusing capacity for carbon monoxide (C). VC; vital capacity, FEV1; forced expiratory volume in 1 second, %DlCO; percentage diffusing capacity for carbon monoxide

The preoperative vital capacity (VC) was 3.6L, the forced expiratory volume in one second (FEV1) was 2.7L, the percentage diffusing capacity for carbon monoxide (%DLCO) was 58.6％ and the lung function predicted by resected lung volume was -26% compared to the preoperative level [4]. A lung function assessment at the time of discharge showed a -54% (1.6L) decrease in VC, -52% (1.3L) decrease in FEV1 and a -78% (12.6%) decline in the %DLCO. Similarly, the six-minute walk distance (6MWD) showed a marked decrease from 450 m to 90 m. Grip strength decreased from 43.7 kg to 21.3 kg, and knee extension strength decreased from 137 Nm to 52 Nm, indicating a substantial decrease in exercise capacity, as well as upper and lower limb strength. By POD 76, weight had decreased from 67.1 kg to 53.0 kg, but supplemental oxygen therapy was introduced, and the case was discharged with a feeling of dyspnea that remained the same. After discharge from the hospital, the case was living independently in self-care, but instrumental activities of daily living required family support. At the 18-month follow-up, VC was -46% (1.9L), FEV1 was -36% (1.71L), %DLCO was -52% (28.3%), grip strength was 27.0 kg, and knee extension strength was 89 Nm, showing improvements, but 6MWD was still 90 m (Table 1, Figure 3).

**Table 1:** Physical capacity evaluations Measured values (pre-operative ratio; %)

	Pre-operative evaluation	Evaluation at discharge	Evaluation after 18 months
6-minute walk distance (m)	450	90 (-80.0)	90 (-80.0)
Grip strength (kg)	43.7	21.3 (-52.3)	27.0 (-38.2)
Knee extension strength (Nm)	137	52 (-62.0)	89 (-35.0)

## Discussion

While the prognosis for acute exacerbation of IP after lung cancer resection is poor, the clinical course for survivors and the types of rehabilitation provided have not been reported. The present case had an acute exacerbation of IP after VATS lobectomy, with a marked worsening of dyspnea and decreased knee extension strength and exercise capacity. While the predicted decline in postoperative lung function was -26%, actual lung function decreased by -54% in VC, -52% in FEV1, and -78% in DLCO, which seemed to cause the worsening dyspnea. Furthermore, it seems obvious that weight loss and muscle weakness due to increased protein catabolism are factors influencing the decline in exercise capacity, in addition to decreased pulmonary function. We have previously evaluated the physical function of patients introduced to supplemental oxygen therapy after VATS lobectomy and reported a 6MWD of -19% and a knee extension strength of -0.3%, compared before and after surgery [5]. In this case, the significant decline in physical function was highlighted by decreases in 6MWD by -80% and knee extension strength by -52.3%. In addition, patients with IP have high levels of anxiety and depression [6], and consideration should be given to the possibility that significant changes in physical function after surgery may exacerbate these symptoms. However, due to the effectiveness of rehabilitation, the current patient was able to maintain their ability to perform basic daily activities and was discharged after a step-by-step intervention. Careful consideration was given to dyspnea and exercise load while also adjusting the type of oxygen administration device.

Both lung function and knee extension strength showed improvements at the 18-month follow-up. On the other hand, the 6MWD was unchanged from the time of discharge, and exercise capacity remained markedly reduced. Miyajima et al. [7]. reported a five-year survival rate of 22.0% after lung cancer resection in patients with acute exacerbation of IP that could be discharged from the hospital. In other words, if acute exacerbation of IP occurs after lung cancer resection, the long-term prognosis is poor even if the patient is discharged from the hospital. In addition, 6MWD is associated with prognosis in patients with IP [8]. Even after long-term follow-up, the lack of improvement in 6MWD may be related to poor prognosis in this case. Continued follow-up will be necessary. In addition, it is necessary to investigate the effect of rehabilitation combined with supplemental oxygen therapy in cases of acute exacerbation of IP after VATS lobectomy in a larger number of patients.

## Conclusions

We experienced a case of acute exacerbation of IP after VATS lobectomy. Step-by-step rehabilitation with oxygen administration commenced as soon as possible, and the patient was able to be discharged. However, physical function and pulmonary function decreased markedly. Eighteen months later, pulmonary function and knee extension strength showed improvements, but exercise capacity was unchanged from the time of discharge. The lack of improvement in 6MWD, even after long-term follow-up, may be related to poor prognosis in this case.
